# Lack of TRIC‐B dysregulates cytoskeleton assembly, trapping β‐catenin at osteoblast adhesion sites

**DOI:** 10.1111/febs.17399

**Published:** 2025-01-20

**Authors:** Barbara Maria Contento, Nadia Garibaldi, Alessandra Sala, Erika Palladino, Amanda Oldani, Alessandra Carriero, Antonella Forlino, Roberta Besio

**Affiliations:** ^1^ Department of Molecular Medicine, Biochemistry Unit University of Pavia Italy; ^2^ Optical Microscopy Facility, Centro Grandi Strumenti University of Pavia Italy; ^3^ Department of Biomedical Engineering The City College of New York NY USA

**Keywords:** cell junctions, cytoskeleton, osteogenesis imperfecta, *TMEM38B*, TRIC‐B, β‐catenin

## Abstract

The trimeric intracellular cation channel B (TRIC‐B), encoded by *TMEM38B*, is a potassium (K^+^) channel present in the endoplasmic reticulum membrane, where it counterbalances calcium (Ca^2+^) exit. Lack of TRIC‐B activity causes a recessive form of the skeletal disease osteogenesis imperfecta (OI), namely OI type XIV, characterized by impaired intracellular Ca^2+^ flux and defects in osteoblast (OB) differentiation and activity. Taking advantage of the OB‐specific *Tmem38b* knockout mouse (*Runx2Cre;Tmem38b* ^*fl*/*fl*^; cKO), we investigated how the ion imbalance affects the osteogenetic process. We found an abnormal cytoskeleton in the cKO OBs, with actin accumulation at OB adhesion sites. The reduced amount of active Ca^2+^‐dependent actin‐binding proteins myristoylated alanine‐rich C‐kinase substrate (MARCKS) and fascin, which modulate cytoskeletal actin dynamics, explains the altered cytoskeletal assembly. The actin clusters at adhesion sites trap β‐catenin, a key structural protein at cell–cell junction sites, that abnormally accumulates despite the significant reduction in both N‐ and E‐cadherins. Besides its structural fuction at cell borders, β‐catenin also has a pivotal role as a transcription factor for proper osteoblastogenesis. Immunofluorescence of cKO nuclei revealed impaired nuclear β‐catenin translocation, further validated in human fetal OB knocked out for *TMEM38B*, which was not rescued by specifically stimulating the canonical Wnt pathway. Thus, we demonstrated *in vitro* that alterations of intracellular Ca^2+^ homeostasis, as a consequence of lack of TRIC‐B, cause cytoskeleton disorganization in cKO OBs, resulting in abnormal β‐catenin accumulation at cell adhesion sites and reduced nuclear β‐catenin translocation, contributing to impaired osteoblastogenesis.

## Graphical abstract

In the presence of TRIC‐B, cytosolic Ca^2+^ activates protein kinase C, which phosphorylates the actin‐binding proteins fascin and MARCKS, leading to proper cytoskeletal organization. This allows an appropriate β‐catenin distribution inside the cell and its translocation into the nucleus, where it stimulates the transcription of osteoblastogenic genes. Lack of TRIC‐B causes impaired intracellular Ca^2+^ handling, compromising the organization of the cytoskeleton, which traps β‐catenin and prevents it from translocating into the nucleus, with a negative impact on osteoblastogenesis. Created with BioRender.com.

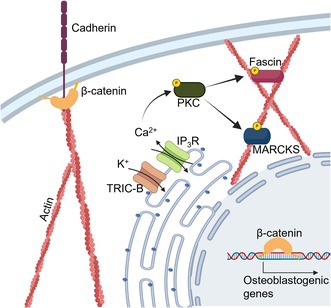

**Abbreviations**
BSA: bovine serum albumin
CaMKII: Ca^2+^‐calmodulin kinase II
cKO: conditional knockout
hFOB: human fetal osteoblasts
DAPI: 4′,6‐diamidino‐2‐phenylindole
ECM: extracellular matrix
FAK: focal adhesion kinase
FBS: fetal bovine serum
IF: immunofluorescence
INM: inner nuclear membrane
IP_3_R: inositol triphosphate receptor
IRM: interference reflection microscopy
OB: osteoblast
OI: osteogenesis imperfecta
PBS: phosphate buffer saline
PFA: paraformaldehyde
pFAK: phospho FAK
pFascin: phospho Fascin
PKC: protein kinase C
pMARKS: phospho MARKS
rER: rough endoplasmic reticulum
SD: standard deviation
SERCA: sarco‐endoplasmic reticulum calcium ATPase
TIRF: total internal reflection fluorescence microscopy
TMEM38B: transmembrane protein 38B
TRIC‐B: trimeric intracellular cation channel B
WNT: wingless‐related integration site
α‐MEM: minimum essential Eagle medium with α‐modification

## Introduction

A fine‐tuned intracellular calcium (Ca^2+^) homeostasis is fundamental for cells and its alteration causes several diseases [1]. Within the cell, the rough endoplasmic reticulum (rER) is the main Ca^2+^ reservoir, and rER channels and SERCA pump play a key role in maintaining the correct Ca^2+^ balance [2]. Among them, the trimeric intracellular cation channel B (TRIC‐B) is a potassium (K^+^) channel that counterbalances inositol triphosphate receptors (IP_3_R)‐dependent Ca^2+^ release from the rER, thus contributing to the regulation of intracellular Ca^2+^ flux and to the maintenance of electroneutrality across the membrane [3, 4]. Mutations in *TMEM38B*, encoding TRIC‐B, impair the rER Ca^2+^ flux and cause the recessive osteogenesis imperfecta (OI) type XIV (OMIM #615066) [5]. OI, or brittle bone disease, is a family of rare inherited collagenopathies characterized by the production of type I collagen molecules with an altered structure and/or reduced collagen secretion and incorporation into the bone extracellular matrix (ECM). Clinical hallmarks of individuals with OI are low bone mass, bone fragility, and skeletal deformities, for the most associated with some extra skeletal phenotypes involving mainly cardiac and respiratory systems [6]. The impaired rER Ca^2+^ flux in absence of TRIC‐B is associated with the synthesis of collagen molecules exhibiting decreased levels of post‐translational modifications. Indeed, a decreased collagen helical hydroxylation of specific lysine residues, carried out by the calcium‐dependent lysine hydroxylase 1, has been demonstrated in OI type XIV probands [7]. Moreover, in an OI type XIV patient, impairment in several cellular functions has been recently observed, such as defects in cell adhesion processes and mitochondrial function, which negatively influence cell proliferation and cycle progression [8]. To bypass the limited availability of OI human bone samples, animal models of the disease are needed. Tric‐b mutant zebrafish revealed an impaired osteoblast and osteoclast activity, together with decreased mineralization, in caudal fin regeneration [9], while the human fetal osteoblasts (hFOB) 1.19 cell line knockout for *TMEM38B* showed reduced osteoblastogenesis markers expression, compromised proliferation and impaired type I collagen structure and secretion [10]. Commonly, mice are the most used animals in bone research for modeling human disease mechanisms since bone morphology, biomechanics, and function are comparable between humans and mice [11]. A global knockout mouse model for *Tmem38b* (*Tmem38b* ^−/−^) was generated, but unfortunately *Tmem38b* ^−/−^ mice died shortly after birth because of respiratory failure [12, 13]. To unravel TRIC‐B function in osteoblasts (OBs), a mouse model with the conditional inactivation of *Tmem38b* by Cre recombinase under the *Runx2* promoter (*Runx2Cre;Tmem38b* ^*fl*/*fl*^, cKO) has been recently generated by our group. The presence in the cKO mice of severe skeletal defects typical of OI, the synthesis of under modified type I collagen and the impairment in osteoblastogenesis, proved the cKO mouse model to be an ideal tool for the investigation of the key roles of TRIC‐B in OB function. In particular, we previously demonstrated that the intracellular Ca^2+^ imbalance causes an impairment in Ca^2+^‐calmodulin kinase II (CaMKII)‐mediated SMAD activation in cKO OBs [14].

Here, we take advantage of the cKO mouse model and tackle the impact of the altered Ca^2+^ homeostasis on the osteogenetic process. We demonstrate that, as a consequence of lack of TRIC‐B, the impaired intracellular Ca^2+^ handling leads to a compromised function of actin‐bundling proteins. As a consequence, a severe compromised cytoskeleton is assembled with actin‐bundling molecules accumulating at cell adhesion sites and trapping β‐catenin that does not translocate into the nucleus where physiologically acts as a transcription factor.

## Results

### Lack of TRIC‐B compromises cell adhesion sites and cytoskeletal organization

**Fig. 1:**
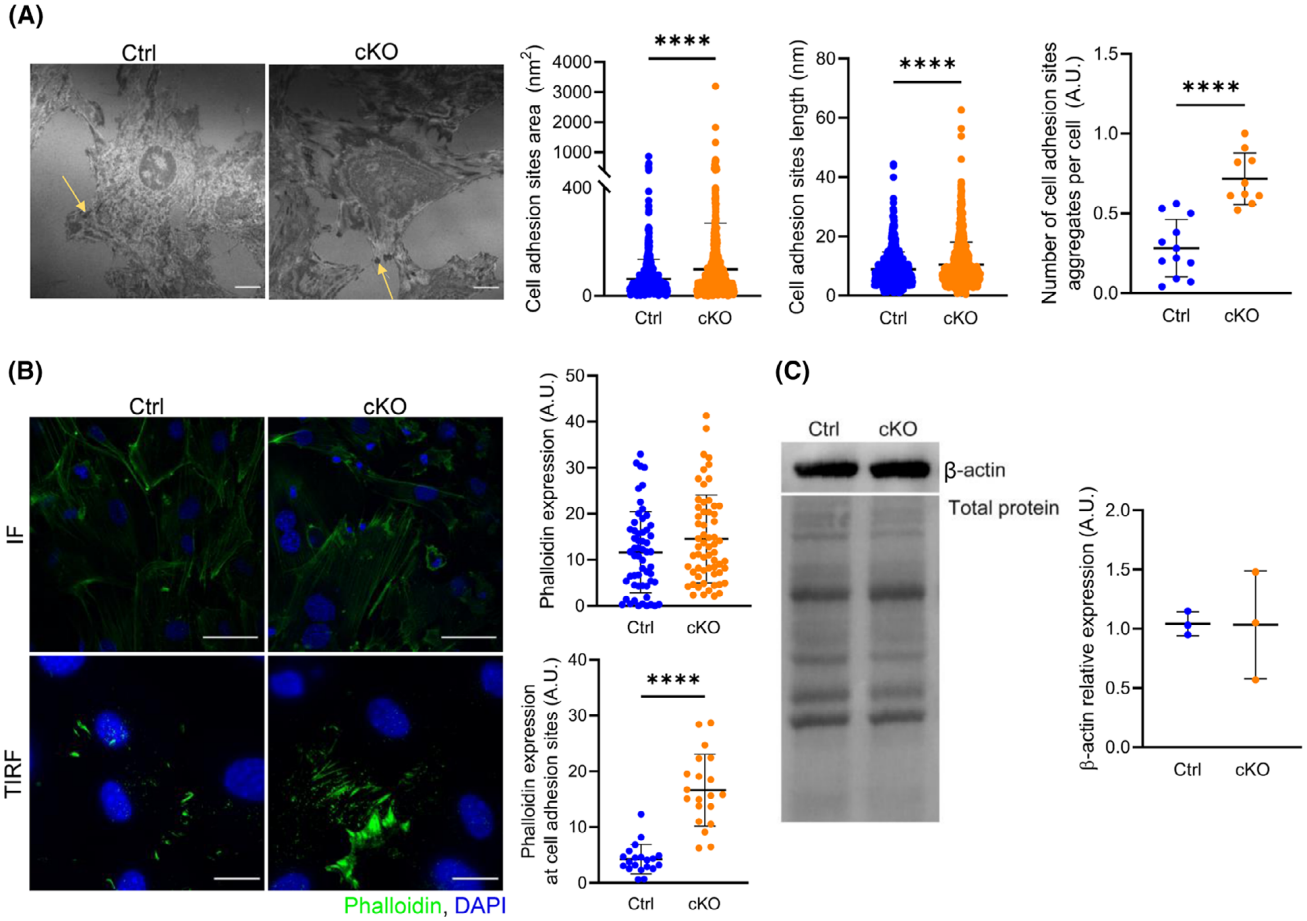
Lack of TRIC‐B compromises osteoblast adhesion sites and cytoskeletal organization. (A) Interference reflection microscopy showed an altered morphology of adhesion sites (yellow arrows), with increased adhesion site area and length with a clustered distribution in osteoblast (OB)‐specific *Tmem38b* knockout mouse (*Runx2Cre;Tmem38b* ^*fl*/*fl*^; cKO) OBs (*n* = 3). Scale bar: 20 μm. (B) Immunofluorescence (IF) analysis of phalloidin, an F‐actin marker, revealed no difference in actin expression between cKO and control OBs, but an abnormal accumulation was found at cKO OBs adhesion sites by total internal reflection fluorescence microscopy (TIRF) (*n* = 3). Scale bar: 20 μm. (C) Western blot analysis confirmed an unchanged level of β‐actin in cKO OBs (*n* = 3). Error bars indicate standard deviation. Student's *t*‐test ****P < 0.0001.

Given the osteoblast impairment detected in absence of TRIC‐B and the relevance of cell–cell connections for the regulation of osteoblastogenesis [15, 16], a morphological analysis of cell adhesion sites was performed by interference reflection microscopy in primary OBs isolated from *Runx2Cre;Tric‐mem38b* ^*fl*/*fl*^ (cKO) mice and their control (*Tmem38b* ^*fl*/*fl*^) littermates. An increased adhesion site area and length, and an increased number of adhesion site aggregates were found in cKO OBs (Fig. 1A), indicating an uneven distribution of adhesion sites with a clustered pattern. The actin cytoskeleton is strictly connected to the cell borders. Thus, the cytoskeletal organization was investigated by analyzing the actin distribution both by immunofluorescence and by total internal reflection fluorescence imaging, the latter allowing the selective excitation of the surface‐bound fluorophores and, thus, the visualization of the cell adhesion sites. In the presence of unchanged levels of total actin, an abnormal cytoskeleton was found in cKO with actin accumulation at OB adhesion sites (Fig. 1B,C).

### Actin‐binding protein impairment is responsible for the cytoskeletal disorganization in *Tmem38b* cKO OBs

**Fig. 2:**
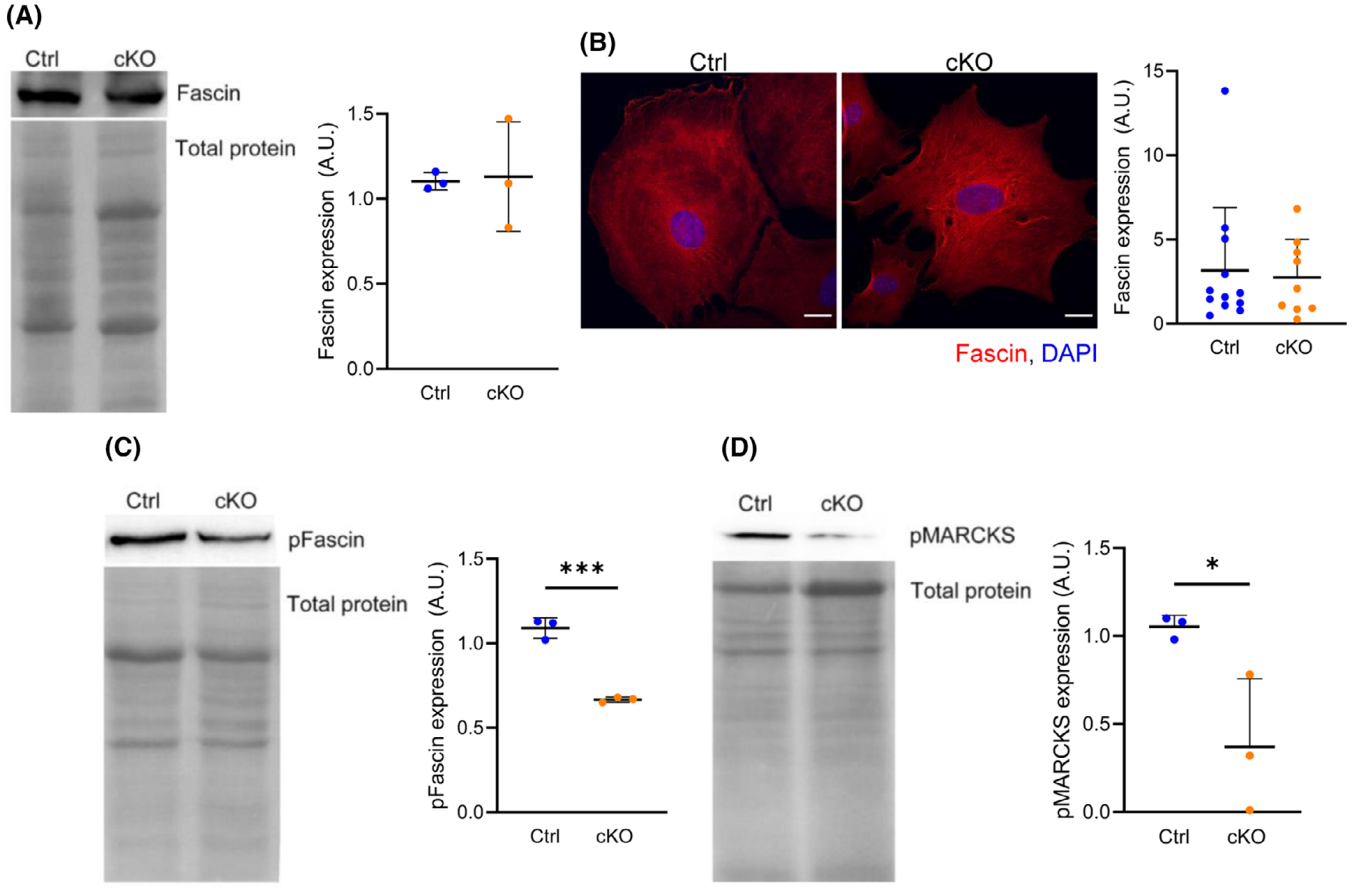
Ca^2+^‐dependent actin‐binding protein impairment causes cytoskeletal disorganization in *Tmem38b* cKO osteoblasts. The activation of the Ca^2+^‐dependent actin‐binding proteins fascin and MARCKS, that bind to actin filaments in order to properly organize their structure and function, was analyzed. (A) Western blot analysis for fascin showed no difference between osteoblast (OB)‐specific *Tmem38b* knockout mouse (*Runx2Cre;Tmem38b* ^*fl*/*fl*^; cKO) OBs and control cells (*n* = 3). (B) Fascin immunofluorescence analysis confirmed the equal expression of fascin in cKO and control cells (*n* = 3). Scale bar: 20 μm. (C) A significant reduction of pFascin (Ser39) indicated an impaired protein activation in cKO OB cells (*n* = 3). (D) A decreased expression of pMARCKS (Ser152/156) was found by western blot analysis in cKO OBs compared to controls (*n* = 3), indicating an impairment of this cytoskeletal regulating protein. Error bars indicate standard deviation. Student's *t*‐test *P < 0.05, ***P < 0.001.

Several proteins bind to actin filaments in the cytoskeleton in order to properly organize their structure and function [17]. Among them, fascin is an actin‐bundling protein, while the myristoylated alanine‐rich C‐kinase substrate (MARCKS) binds to F‐actin at plasma membrane [18, 19]. These proteins' activity is modulated by Ca^2+^‐activated conventional isoforms of protein kinase C (PKC) [20, 21]. In the presence of a normal amount of nonphosphorylated protein (Fig. 2A,B), a reduced amount of pFascin (Ser39) was found in cKO OBs (Fig. 2C), suggesting an impairment in the mutant cell cytoskeletal dynamics. Upon phosphorylation by PKC, MARCKS translocates from the membrane into the cytosol, modulating cytoskeletal actin dynamics. Mutant OBs showed decreased levels of pMARCKS (Ser152/156) (Fig. 2D), indicating a possible MARCKS accumulation that can compromise the actin cytoskeleton organization at cell adhesion sites. Thus, the intracellular Ca^2+^ homeostasis impairment consequent to a lack of TRIC‐B negatively affects the actin cytoskeleton organization through Ca^2+^‐dependent actin‐binding proteins and indirectly influences the cell adhesion sites organization.

### Adhesion site disorganization impairs cell‐matrix focal adhesion in *Tmem38b* cKO OBs

**Fig. 3:**
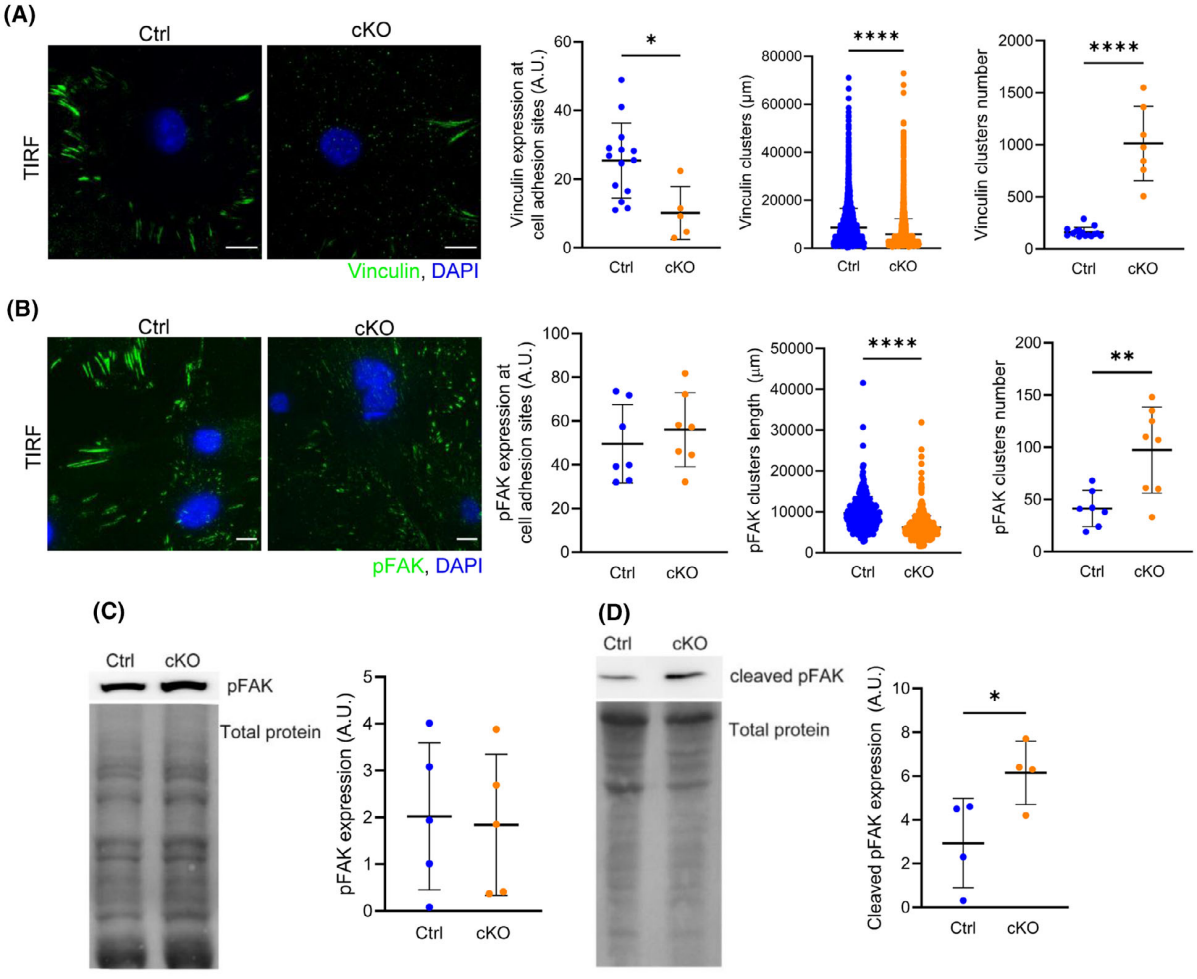
Actin cytoskeletal disorganization impairs cell–matrix focal adhesion in *Tmem38b* cKO osteoblasts. (A) Interference reflection microscopy (TIRF) imaging for vinculin, an actin crosslinking protein important for cytoskeletal integrity, which connects cytoskeleton to cell–cell and cell–extracellular matrix junctions, revealed a decreased vinculin expression at (OB)‐specific *Tmem38b* knockout mouse (*Runx2Cre;Tmem38b* ^*fl*/*fl*^; cKO) adhesion sites, and also a vinculin fragmentation in cKO with an increased number of shorter clusters (*n* = 3). Scale bar: 100 μm. (B) TIRF imaging of the activated form of the focal adhesion kinase (FAK), a key player of the focal adhesion complex that regulates the actin cytoskeleton organization, showed no difference in the pFAK level between cKO and control but revealed an altered distribution of the phosphorylated protein with an increased number of shorter pFAK clusters in cKO OBs (*n* = 3). Scale bar: 100 μm. (C, D) Western blot analyses indicated an increased pFAK fragmentation in presence of unchanged levels of not cleaved protein in cKO OBs compared to controls (*n* = 3). Error bars indicate standard deviation. Student's *t*‐test *P < 0.05, **P < 0.001, ****P <0.0001.

Given the impairments at cell borders, the *Tmem38b* cKO OB cell‐ECM connections were here analyzed. In particular, the focal adhesion kinase is a key player of the focal adhesion complex, as it regulates the structure of the cell adhesion sites and the actin cytoskeleton organization [22]. Within the focal adhesion complex, vinculin is pivotal, as upon binding to actin, it stimulates actin polymerization [23]. A reduced and differentially organized vinculin was detected in cKO OBs (Fig. 3A) and was associated with an increased number of shorter vinculin clusters.

Another important player in the focal adhesion complex is the focal adhesion kinase (FAK), which autoactivates through auto‐phosphorylation at Tyr397, and transduces extracellular stimuli in intracellular responses [22]. Thus, we analyzed FAK activation by TIRF analysis, which revealed an increased number of shorter pFAK clusters at the mutant OB adhesion sites, with the same overall amount of pFAK (Fig. 3B). Moreover, no differences were found in total pFAK expression, but an increased level of cleaved pFAK was detected by western blot in mutant cells (Fig. 3C,D). Taken together, these data indicate affected cell adhesion sites at cell‐ECM level in cKO OBs.

### Abnormal actin cytoskeleton causes β‐catenin accumulation at *Tmem38b* cKO cell adhesion sites

**Fig. 4:**
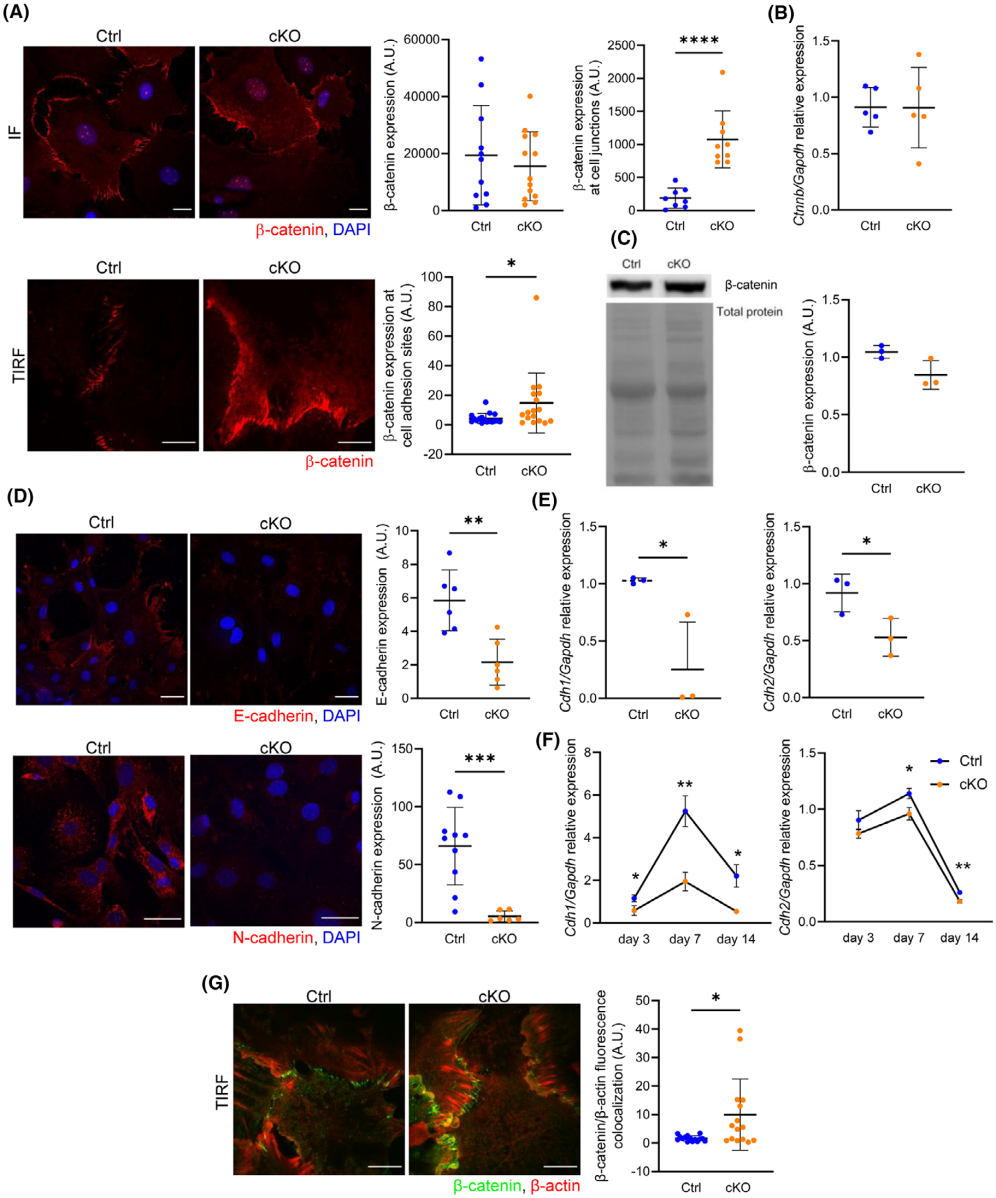
Abnormal β‐catenin accumulation at *Tmem38b* cKO osteoblast adhesion sites in absence of cadherins. (A) In presence of unchanged levels of total β‐catenin, an increased junctional β‐catenin was found in (OB)‐specific *Tmem38b* knockout mouse (*Runx2Cre;Tmem38b* ^*fl*/*fl*^; cKO) OBs by immunofluorescence (IF) and confirmed by total internal reflection fluorescence (TIRF) (*n* = 4). Scale bar: 20 μm. (B) Gene expression analysis for β‐catenin (*Ctnnb*) showed no change between cKO and control cells (*n* = 3). (C) Western blot analysis confirmed no difference in β‐catenin protein expression between Ctrl and cKO OBs (*n* = 3). (D) Immunofluorescence analyses revealed a reduced N‐ and E‐cadherin expression in cKO OBs compared to controls (*n* = 3). Scale bar: 40 μm. (E) A reduced N‐ and E‐cadherin expression was also found at mRNA level by RT‐qPCR both at steady state and (F) during cell mineralization (*n* = 3). (G) TIRF double labeling for β‐catenin and β‐actin showed an increased colocalization at cKO OBs adhesion sites indicating that β‐actin traps β‐catenin at cell adhesion sites (*n* = 3). Scale bar: 20 μm. Error bars indicate standard deviation. Student's *t*‐test *P < 0.05, **P < 0.001, ***P < 0.0001, ****P < 0.0001.

Besides cell‐ECM connections, the cell–cell junctions are essential protein complexes for OB differentiation, and β‐catenin represents, together with cadherins, an important structural player since it connects the cell–cell junctions to the actin cytoskeleton. Indeed, newly synthesized β‐catenin can be either immobilized at adherens junctions by cadherins or, to a lesser extent, be kept into the cytosol [24]. Unchanged level of total β‐catenin was found at the protein level and supported at the gene expression level by RT‐qPCR analyses (Fig. 4A–C). Interestingly, analyses of the β‐catenin cellular localization by confocal microscopy revealed that the protein highly accumulates at *Tmem38b* cKO cell junctions (Fig. 4A). Total internal reflection fluorescence, highlighting the cell adhesion sites, confirmed this observation (Fig. 4A).

Given the β‐catenin strict link with the cell–cell junction cadherins, their expression levels were assessed. Surprisingly, a significant reduction in both N‐ and E‐cadherin protein levels was found in the cKO OBs (Fig. 4D), confirmed also at the transcriptional level both at steady state and during cell mineralization (Fig. 4E,F). Importantly, β‐catenin mainly colocalized with actin aggregates at cell adhesion sites (Fig. 4G). Thus, the abnormal actin cytoskeleton deposition at mutant cell adhesion sites traps β‐catenin, causing its accumulation even in absence of cadherins.

### Reduced β‐catenin nuclear internalization in absence of TRIC‐B

**Fig. 5:**
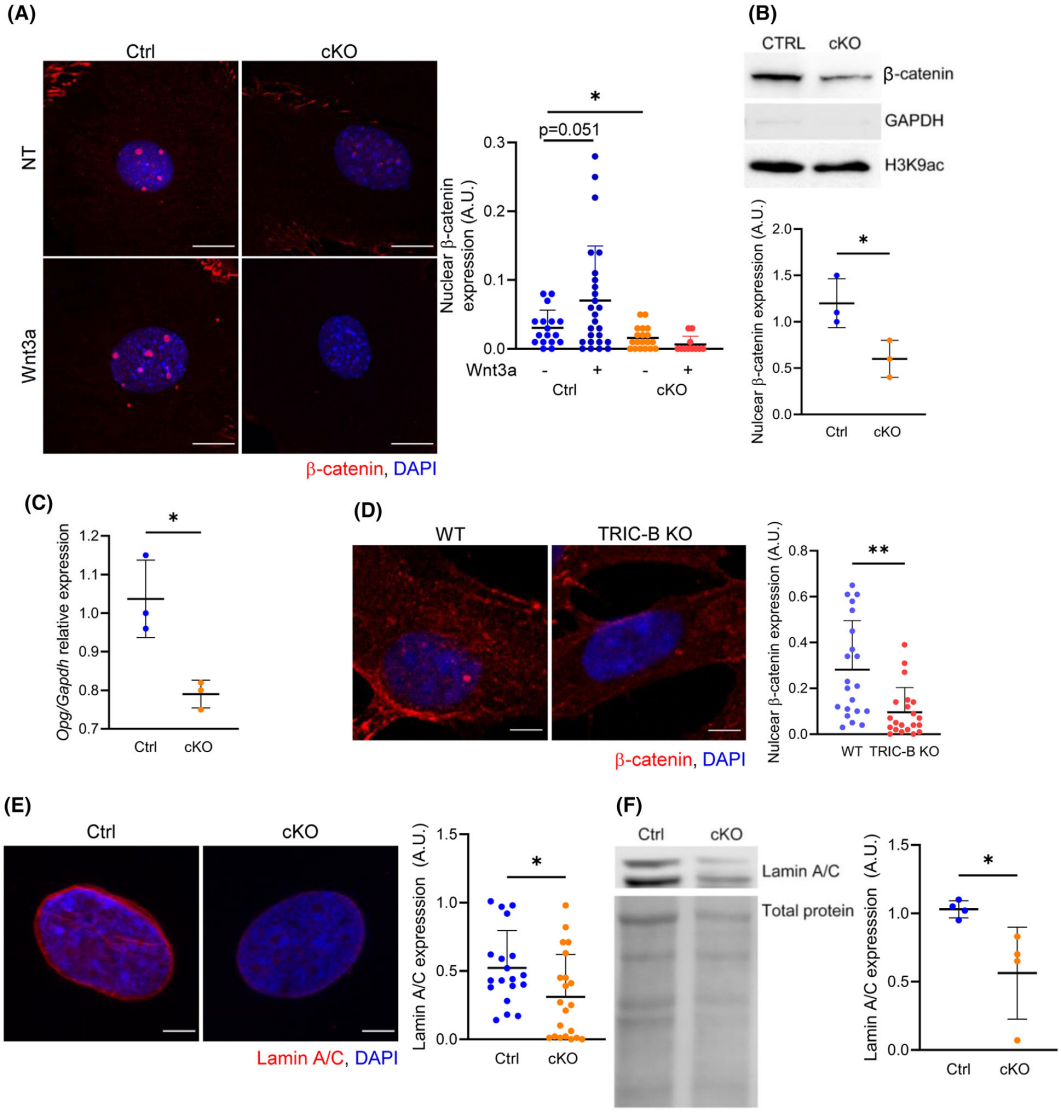
Reduced β‐catenin nuclear internalization in absence of TRIC‐B. (A) Immunofluorescence analyses of β‐catenin showed a decrease in β‐catenin nuclear content in (OB)‐specific *Tmem38b* knockout mouse (*Runx2Cre;Tmem38b* ^*fl*/*fl*^; cKO) OBs compared to controls (*n* = 3). Treatment with recombinant Wnt3a, to stimulate β‐catenin nuclear internalization, effective on control cells, had no effect on cKO OBs. Scale bar: 40 μm. (B) A reduced nuclear β‐catenin level was confirmed by western blot analysis on proteins of the nuclear fraction (*n* = 3). (C) RT‐qPCR analysis revealed a reduced expression of *Opg* in cKO OBs compared to controls (*n* = 3). (D) Immunofluorescence analyses for β‐catenin in human fetal OBs confirmed the reduced β‐catenin nuclear translocation in *Tmem38b* KO cells compared to wild‐type (WT) cells (*n* = 3). Scale bar: 5 μm. (E) Immunofluorescence analyses of lamin A/C, a component of the inner nuclear membrane indirectly involved in β‐catenin nuclear localization, were carried out. Lamin A/C expression was found reduced in cKO OBs compared to controls (*n* = 3). Scale bar: 5 μm. (F) The reduced lamin A/C expression in mutant cells was confirmed by western blot analysis (*n* = 3). Error bars indicate standard deviation. Student's *t*‐test *P < 0.05, **P < 0.001.

Besides its structural role at the cell borders, β‐catenin has a pivotal role for promoting proper osteoblastogenesis in association with the TCF/Lef family of transcription factors. Indeed, it is known that a correct intracellular Ca^2+^ homeostasis is important for the β‐catenin nuclear translocation [25] and that IP_3_‐signaling plays an important role in regulating the nucleoplasmic Ca^2+^ concentration [26]. Thus, we assessed the nuclear β‐catenin localization. Immunofluorescence of the cKO OB nuclei revealed an impaired nuclear β‐catenin translocation (Fig. 5A,B). We evaluated the nuclear translocation also following cell incubation with 200 ng·mL^−1^ recombinant Wnt3a, the main ligand stimulating the canonical Wnt pathway. The treatment showed a positive effect on the control cells, while no rescue was found in the cKO OBs, suggesting a compromised Wnt pathway (Fig. 5A). The reduced expression of *Opg* (Fig. 5C), a target gene of β‐catenin TCF/Lef complex [27], supported our findings. A decreased nuclear β‐catenin amount was also confirmed in human fetal OBs (hFOB) knocked out for *Tmem38b* (Fig. 5D). Besides Ca^2+^, also lamin A/C, in the inner nuclear membrane, is indirectly involved in the β‐catenin nuclear localization [28, 29]. Of relevance, a decreased amount of lamin A/C was found in the cKO OBs (Fig. 5E,F), likely contributing to the impaired translocation.

## Discussion

**Fig. 6:**
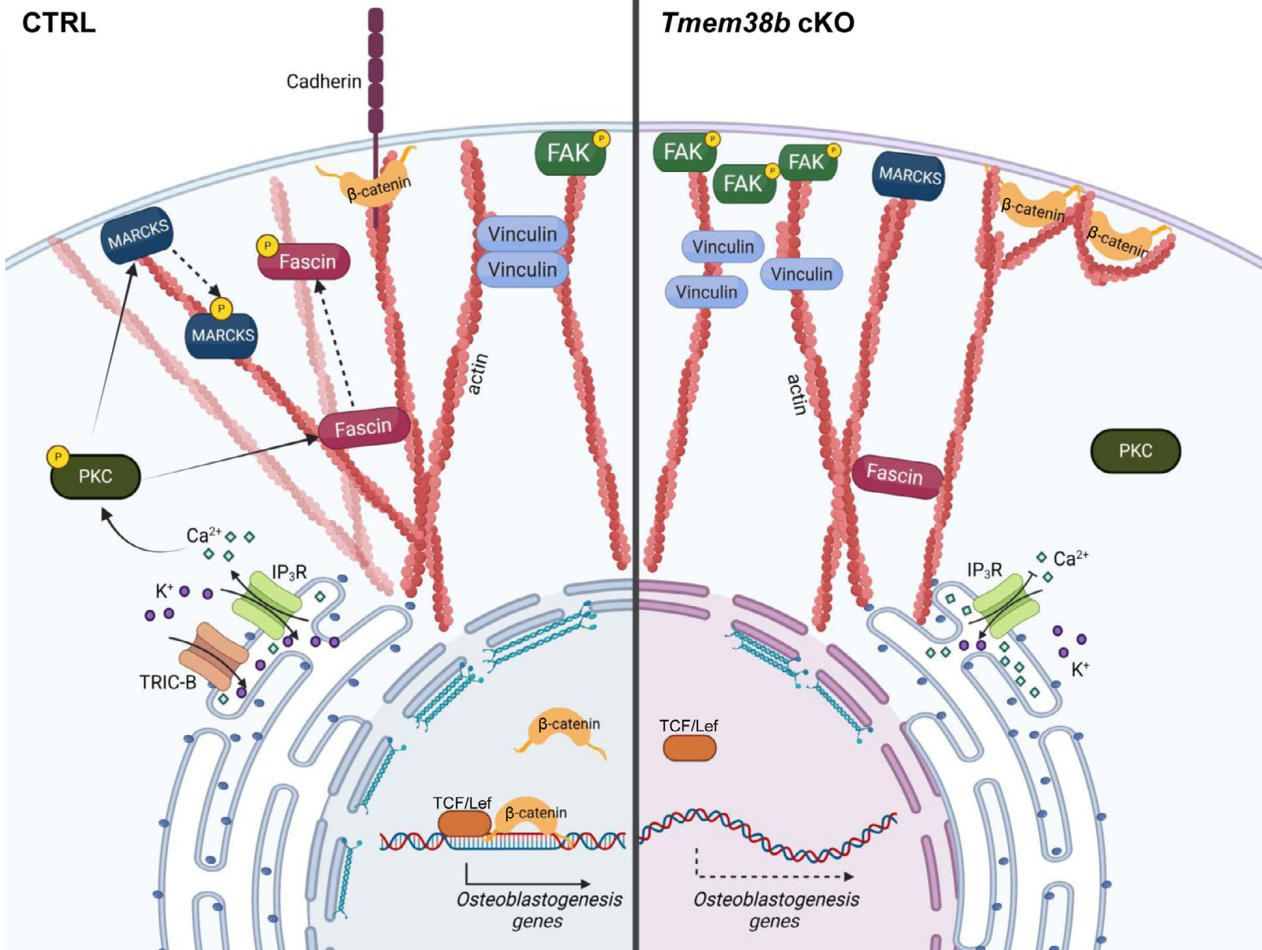
Schematic view of the mechanisms of β‐catenin mislocalization in *Tmem38b* cKO OBs. In control OBs (CTRL), the presence of TRIC‐B, encoded by *Tmem38b*, on the endoplasmic reticulum membrane allows a physiological intracellular Ca^2+^ flux through the IP_3_R channel that activates the Ca^2+^‐dependent PKC. PKC, in turn, activates through phosphorylation the actin‐binding proteins fascin and MARCKS. As a response, actin cytoskeleton arranges in an ordered network allowing a proper activation of cell–extracellular matrix focal adhesion complex (FAK) and a proper spatial organization of the actin crosslinking protein vinculin and of cell–cell β‐catenin/cadherins complex. Free β‐catenin can translocate inside the nucleus, also thanks to the nucleoskeleton filaments lamin A/C, allowing the transcription of osteoblastogenic genes. In *Tmem38b* cKO OBs, the absence of a functional TRIC‐B channel causes an impaired intracellular Ca^2+^ handling. This leads to a reduced activation of the PKC and, as a consequence, to a disorganization of the actin cytoskeleton that is reflected into altered cell connections. The abnormal actin aggregates at cell adhesion sites trap β‐catenin, causing its accumulation even in absence of cadherins. As a result, β‐catenin does not translocate into the nucleus to allow the transcription of genes important for osteoblastogenesis, explaining the delay in osteoblastogenesis that characterizes mutant OBs. Created with BioRender.com.

In the present study, we demonstrated *in vitro* that alteration of the intracellular Ca^2+^ homeostasis, as a consequence of lack of TRIC‐B, causes a cytoskeleton and nucleoskeleton disorganization in cKO OBs, resulting in an abnormal β‐catenin accumulation at cell adhesion sites and in a reduced nuclear β‐catenin translocation, contributing to impaired osteoblastogenesis (Fig. 6).

Bone is a dynamic tissue that constantly remodels by the coordinated activities of osteoblasts, osteoclasts, and osteocytes, thanks to a continuous cell–cell communication [30]. In this context, cell–cell and cell–ECM interactions are fundamental for a proper bone homeostasis, allowing cell communication and sensing of the extracellular environment. Several types of cell connections have been described in bone [31]. The process of cell adhesion is regulated by Ca^2+^ ions, which are present both in the intracellular and extracellular compartments. At the extracellular level, four Ca^2+^ ions mediate the adhesion between cadherins in the extracellular space [32]; while intracellularly, Ca^2+^ modifies cell polarity through cytoskeletal actin fibers influencing the cell adhesion mechanisms, as demonstrated by Adachi *et al*. [33] in WT OBs being mechanically stimulated. Thus, the cytoskeleton disorganization and the accumulation of actin at OB adhesion sites and at the aggregated cell adhesion sites in mutant cells are likely explained by the altered Ca^2+^ flux inside the cKO cells. Of relevance, defects in cytoskeletal organization have already been observed in dominant and recessive OI patients, as well as in the Brtl^+/−^ mouse model of OI [34, 35], although without assessing the mechanisms. Also, dysregulations in proband fibroblasts vimentin, stathmin, and cofilin‐1, which are proteins functionally linked to the cytoskeleton, were found and their expression directly correlated with the severity of the OI phenotype [36]. Cytosolic Ca^2+^ levels also influence some actin‐binding proteins, acting directly on proteins or indirectly through Ca^2+^‐dependent kinases like the classical isoforms of protein kinase C (PKC) [37, 38]. This contributes to explain the abnormal actin cytoskeletal organization at cell adhesion sites since both fascin and the myristoylated alanine‐rich C‐kinase substrate (MARCKS), whose activation is impaired in cKO OBs, are actin‐binding proteins regulated by PKC [21, 39]. Indeed, fascin phosphorylation at Ser39 or Ser274 is the major mechanism that inhibits its actin‐bundling activity [20], while, upon phosphorylation, MARCKS translocates from the membrane into the cytosol, modulating cytoskeletal actin dynamics and vesicular trafficking and activating various signal transduction pathways [40]. Notably, a previous link has been described between the alteration of a Ca^2+^‐activated cytoskeletal protein, plastin 3, and OI [41].

Cell‐ECM connections, such as the focal adhesion complex, are important in OBs. The focal adhesion complex connects the extracellular environment to the actin cytoskeleton through different proteins and regulates cellular mechanisms linked to spreading, migration, differentiation, and mechanotransduction [42]. Its alterations cause bone‐related pathologies, such as osteoporosis and osteoarthritis [43]. The focal adhesion kinase (FAK) is one of the major components of the focal adhesion complex that autophosphorylates at Tyr397 when the integrins attach to the ECM, initiating a signaling cascade [16]. It has been reported that OBs from osteoporotic bone sites exhibit a reduced tyrosine phosphorylation of the FAK and that FAK null cells fail to completely differentiate into osteoblasts [44]. A compromised integrity of the focal adhesion complex is supported by an increased cleavage of phosphorylated FAK, generally caused by ROS‐activated caspases [45]. Indeed, an increased ROS production was recently found in a *TMEM38B* patient [8].

A Ca^2+^‐dependent compromised actin cytoskeleton is supported by the reduced amount of vinculin, an actin crosslinking protein important for cytoskeletal integrity, that connects cell–cell and cell–ECM junctions to the cytoskeleton, and whose incorporation in the cell–matrix focal adhesion complex is influenced by Ca^2+^ [23, 46].

In the 80s, β‐catenin was described as both a structural and a signaling molecule. In particular, Ozawa *et al*. [47] for the first time identified β‐catenin as a structural protein that binds to cell–cell junction molecules, connecting them to the cytoskeleton. Simultaneously, the function of β‐catenin as signaling molecule has been described in *Drosophila melanogaster* [48, 49]. To date, it is well established that β‐catenin is the key nuclear effector of the canonical wingless‐related integration site (Wnt) pathway as well as an integral structural component of cadherin‐based adherens junctions. An imbalance in the β‐catenin structural and signaling properties often results in a disease [24]. As a structural component of the cell–cell cadherin adhesion complex, β‐catenin binds to the cytoplasmic tail of both N‐ and E‐cadherin that limit its free availability [50, 51], but it also serves as a ‘bridge’ that connects the cell–cell junctions to the actin cytoskeleton [32, 52]. Its abnormal accumulation at cKO OB adhesion sites, in presence of normal protein expression, indicates β‐catenin impaired distribution in mutant cells. If from one side, the unexpected, reduced expression of both N‐ and E‐cadherin in cKO OBs is not helping to explain the impaired β‐catenin distribution, from the other side the increased β‐catenin/β‐actin colocalization in cKO OBs indicates that β‐catenin is trapped at the cell junctions by actin cytoskeleton as a consequence of its abnormal accumulation due to impaired Ca^2+^ homeostasis.

As outlined earlier, during the canonical Wnt/β‐catenin cascade, β‐catenin translocases into the nucleus where it associates with transcription factors from the TCF/Lef family, stimulating the transcription of genes important for osteoblastogenesis, such as *Opg* [24, 27]. The reduced nuclear β‐catenin internalization found here in cKO OBs and validated in *TMEM38B* KO human cells, together with a decreased expression of *Opg*, contributes to explain the impaired osteoblastogenesis that characterizes these cells [10, 14]. Indeed, proteomic analysis previously suggested a compromission in the Wnt pathway in a *TMEM38B*‐null patient [8]. The low level of nuclear cKO β‐catenin after treatment with recombinant Wnt3a supports a Wnt canonical pathway impairment. Besides the canonical Wnt/β‐catenin pathway, also the Ca^2+^‐activated noncanonical Wnt signaling influences TCF/Lef activation through the CaMKII [53, 54]. Indeed, a reduced activation of CaMKII has been already reported by our group, given the intrinsic impaired rER calcium handling, indicating a compromised noncanonical Ca^2+^/CaMKII Wnt pathway [14, 53, 54]. Numerous mechanisms are involved in the β‐catenin nuclear localization, and Ca^2+^ has been demonstrated to play a key role in this process [25, 55]. Indeed, IP_3_R‐dependent Ca^2+^ flux is a regulator of the nuclear Ca^2+^ concentration, while Ca^2+^ gradient across the membrane is pivotal for nuclear import of several proteins [25, 26]. Also, the inner nuclear membrane (INM) protein emerin has been demonstrated to stimulate the β‐catenin flux into the nucleus [56] when it localizes at the INM, through its association with the type V intermediate filament proteins and lamin A/C [28, 29]. Thus, the reduction in the expression of lamin A/C in cKO OBs represents a further obstacle for the β‐catenin nuclear translocation [14, 57]. Indeed, lamin A/C expression is dependent on CaMKII activation, known to be altered in absence of TRIC‐B [14].

In conclusion, our work elucidated how an impaired intracellular Ca^2+^ flux impacts on β‐catenin localization, remarking the relevant role of nucleoskeletal and cytoskeletal organization for a proper intracellular β‐catenin distribution.

## Materials and methods

### Animals

C57Bl/6N gray *Tmem38b* ^*fl*/*fl*^ (control), *Runx2Cre;Tmem38b* ^*fl*/*fl*^ (cKO), and FVB/NTg (*Runx2‐Cre*) mice were used in the study [14]. *Tmem38b* ^*fl*/*fl*^ mice were generated by Poly Gene and the FVB/NTg (*Runx2‐Cre*) mice were provided by Prof. Tuckermann (University of Ulm, Germany).

Mice were hosted at ‘Centro interdipartimentale di servizio per la gestione unificata delle attività di stabulazione e di radiobiologia’ of the University of Pavia, Italy. Experiments were performed in accordance with the standard experimental animal care protocol following the Italian Laws D.Lgs. 26/2014 (Animal protocol approval n° 458/2023‐PR).

### Murine calvarial osteoblast culture

Primary murine OBs were isolated from 2 to 4 days old control (*Tmem38b* ^*fl*/*fl*^) and cKO (*Runx2Cre;Tmem38b* ^*fl*/*fl*^) male and female pups, as previously reported [58]. Cells at passage 1 were plated at 1.5 × 10^4^/cm^2^, cultured in minimum essential Eagle medium with α‐modification (α‐MEM) with 10% fetal bovine serum (FBS), 100 mg·mL^−1^ penicillin and streptomycin (Euroclone, Milan, Italy) and 50 μg·mL^−1^ ascorbic acid (Sigma‐Aldrich, St. Louis, MO, USA). For mineralization studies, cells were cultured in a osteogenic medium: α‐MEM with 10% FBS, antibiotics and supplemented with 100 μg·mL^−1^ ascorbic acid and 10 mm β‐glycerophosphate (Sigma‐Aldrich). For recombinant Wnt3a (R&D System, Minneapolis Minnesota, USA) treatment, cells were incubated with 200 μg·mL^−1^ of the ligand for 24 h. All experiments were performed with mycoplasma‐free cells.

### Human fetal osteoblast culture

The immortalized human fetal osteoblast (hFOB) 1.19 A3 clonal line knockout for *TMEM38B* and the not transfected hFOB control (ATCC, CRL‐11372), authenticated in the past 3 years (STR profiling), were used for β‐catenin immunofluorescence experiments [10]. To this purpose, cells were grown at 34 °C in humidified atmosphere containing 5% CO_2_ in growing medium made of Dulbecco's modified Eagle's medium/Nutrient Mixture F‐12 Ham (DMEM:F12) (Sigma‐Aldrich) containing 2.5 mm L‐glutamine and 15 mm 2‐[4‐(2‐hydroxyethyl)piperazine‐1‐yl] ethanesulfonic acid (HEPES) and added with 10% fetal bovine serum (Euroclone) and 0.3 mg·mL^−1^ geneticin (Roche, Basel, Switzerland).

### Gene expression analysis

Total RNA was extracted, with QIAzol Lysis Reagent (Qiagen, Hilden, Germany) according to the manufacturer's protocol, from primary OBs at steady state and after 3, 7, and 14 days of culture in osteogenic medium. E‐cadherin (*Cdh1*), N‐cadherin (*Cdh2*), β‐catenin (*Ctnnb*), and osteoprotegerin (*Opg*) gene expression were analyzed by RT‐qPCR with the QuantStudio 3 thermocycler (Thermo Fisher) using PowerUp Syber Green Master Mix (Applied Biosystems, Waltham, MA, USA). *Gapdh* was used as a normalizer. Relative expression was calculated by the ΔΔ*C*_t_ method. At least biological triplicates for each genotype were performed. Primers are available upon request.

### Total protein lysates and cellular fractioning

Cellular protein lysates were obtained from cultured primary OBs using RIPA lysis buffer (150 mm NaCl, 1% IGEPAL^®^ CA‐630, 0.5% sodium deoxycholate, 0.1% SDS, and 50 mm Tris, pH 8.0) supplemented with protease inhibitors, as previously described [58].

For the cytosolic fraction, cells were lysed in NP‐40 buffer (10 mm Tris/HCl pH 7.9, 140 mm KCl, 5 mm MgCl_2_, 0.5% NP‐40, 10 mm benzamidine, 0.25 mg·mL^−1^ NEM, 4 mm EDTA, 1 mm PMSF, 2 mm sodium orthovanadate) and centrifuged at 1000 ***g*** for 5 min at 4 °C. The supernatant contained the cytosolic fraction. For the nuclear fraction, the pellet was lysed and sonicated in 100 mm Tris/HCl pH 7.4, 0.5% Triton X‐100, 0.5% SDS and then centrifuged at 15 000 ***g*** for 5 min at 4 °C. Proteins were quantified by QuantumProtein Bicinchoninic Protein Assay Kit (Euroclone).

### Western blot analysis

Proteins were separated on a 12% (50 μg for β‐catenin, 40 μg for pFAK) and 10% (50 μg for Fascin, pFascin and 30 μg for pMARCKS) SDS/PAGE and transferred on PVDF membrane. The membranes were incubated o/n at 4 °C with the following antibodies: 1 : 1000 anti‐β‐catenin (Abcam ab32572, Cambridge, UK) in 5% bovine serum albumin (BSA), 1 : 1000 anti‐pFAK (Invitrogen 700255, Waltham, MA, USA) in 5% milk, 1 : 700 anti‐Fascin (Cell Signaling 54545, Danvers, MA, USA) in 5% BSA, 1 : 1000 anti‐pFIascin (Invitrogen PA5‐143700) in 5% BSA, 1 : 1000 anti‐β‐actin (Abcam ab8226) in 5% BSA, 1 : 500 anti‐lamin A/C (Santa Cruz Biotechnology, Dallas, TX, USA) sc‐20681) in 5% milk. ImageQuant LAS 4000 (GE Healthcare, Milan, Italy) software was used for image acquisition. ImageJ was employed for band intensity evaluation. Protein normalization was assessed by total protein staining with Swift™ Membrane Stain (G‐Biosciences, St. Louis, MO, USA). For the cytosolic fraction, the protein loading normalization was determined using anti‐GAPDH antibody (Abcam ab181602) and for the nuclear fraction using an anti‐H3K9ac antibody (Invitrogen, MA5‐11195). For each gel, the expression of the cKO samples was quantified as a fold difference compared to control OBs. At least biological triplicates for each genotype were performed. Original, uncropped images of all western blots are reported in Fig. S1.

### Interference reflection microscopy (IRM)

2 × 10^4^ OBs were plated on sterile glass coverslips (Paul Marienfeld GmbH, Lauda‐Königshofen Germany) in 6‐well plates and cultured for 5 days. Cells were fixed with 4% paraformaldehyde (PFA) for 20 min. Samples in PBS were acquired in bright field using a Leica TCS SP8 confocal microscope (Leica, Wetzlar, Germany) equipped with a 63x oil immersion objective (Leica HC PL APO CS2 63×/1.40). Junctions were measured using the software LAS 4.5 (Leica). For cell adhesion cluster identification, a polygon was used as a mask to define the cluster. For each cell, we checked the number of polygons including the whole cell adhesion. We normalized this number to the total number of cell adhesion sites within the same cell. Cell adhesion site area and length were also quantified. At least biological triplicates for each genotype were performed.

### Total internal reflection fluorescence microscopy (TIRF)

2 × 10^4^ OBs were plated on sterile glass coverslips (Paul Marienfeld GmbH) in 6‐well plates and cultured for 5 days. For phalloidin analyses, cells were fixed in 10% neutral buffered formalin, permeabilized with 0.1% Triton X‐100 in PBS, and incubated with Phalloidin‐iFluor 488 Reagent (Abcam ab176753) in 1% BSA. For vinculin and phosphorylated focal adhesion kinase (pFAK) analyses, cells were fixed with 4% PFA and incubated with anti‐vinculin (Sigma‐Aldrich V4139, 1 : 100) and anti‐pFAK (Thermo Fisher 700255, 1 : 500), respectively. For β‐catenin and β‐actin analyses, cells were fixed in ice‐cold methanol and incubated with anti‐β‐catenin (Abcam ab32572, 1 : 200) and anti‐β‐actin (Abcam ab8226, 1 : 200). 4′,6‐diamidino‐2‐phenylindole (DAPI) was used for nuclei staining. Samples were analyzed in PBS using a DMi8S TIRF microscope (Leica) equipped with a TIRF 100× oil immersion objective (Leica HC PLAN APO 100×/1.47). The fluorescence signal was measured using  ImageJ software. The vinculin and pFAK expression and cluster number were normalized in each cell in order to have a mean cell value. The vinculin cluster length was referred to the length of each cluster present in all the analyzed cells. At least biological triplicates for each genotype were performed.

### Immunofluorescence (IF)

5 × 10^3^ OBs were plated on glass coverslips in 24‐well plate and cultured for 5 days. For phalloidin staining cells were fixed in 4% PFA, permeabilized with 0.1% Triton X‐100 in PBS and incubated with Phalloidin‐iFluor 488 reagent in 1% BSA. For E‐cadherin, cells were fixed in 4% PFA, permeabilized with 0.1% Triton X‐100 in PBS, blocked with 5% BSA, 0.1% Triton X‐100 in PBS and then incubated with anti‐E‐cadherin (Abcam ab231303, 1 : 100). For N‐cadherin, cells were fixed in 4% PFA, blocked with 5% milk in PBS and then incubated with anti‐N‐cadherin (Abcam ab98952, 1 : 200). For murine β‐catenin, cells were fixed in ice‐cold methanol, permeabilized with 0.1% Triton X‐100 in PBS, blocked with 1% BSA 0.1% Tween 20 in PBS and then incubated with anti‐β‐catenin (Abcam ab32572, 1 : 200). For human β‐catenin, cells were fixed in ice‐cold methanol, permeabilized with acetone, and incubated with anti‐β‐catenin (Sigma‐Aldrich c7082, 1 : 1000). For fascin, cells were fixed in 4% PFA, permeabilized with ice‐cold methanol, and incubated with anti‐fascin (Cell Signaling 54545, 1 : 50). For lamin A/C, cells were fixed in ice‐cold methanol, blocked for 1 h in 1% BSA in phosphate buffer saline (PBS), 0.3% Triton X‐100, and then incubated with anti‐lamin A/C (Santa Cruz Biotechnology sc‐20681, 1 : 200). Nuclei were stained with DAPI, and images were acquired by confocal microscope TCS SP8 (Leica). The fluorescence signal was measured using imagej software. At least biological triplicates for each genotype were performed. Negative controls are reported in Fig. S2.

### Statistical analysis

Quantitative variables were expressed as mean ± standard deviation (SD). Statistical differences between cKO and Ctrl OBs or *TMEM38B* KO and WT OBs were evaluated by Student's *t*‐test. A *P* value < 0.05 was considered significant.

## Author contributions

RB and AF: Conceptualization; BMC, NG, AO, AS, AC; EP, and RB: Methodology; BMC, AC AF, and RB: Formal analysis; RB; AF; and AC: Resources; BMC, AF, and RB: Data curation; BMC, AF, and RB: Writing original draft; all the coauthors: Writing—review and editing; RB and AF: Supervision; RB: Project administration; RB; AC; and AF: Funding acquisition.

## Conflict of interest

The authors declare no conflict of interest.

## Supporting information


**Fig. S1.** Original, uncropped images of all western blots.
**Fig. S2**. Negative controls for the immunofluorescence analyses.

## Data Availability

The data that support the findings of this study are available from the corresponding author (roberta.besio@unipv.it) upon reasonable request.
