# Supplementary material for: Lack of TRIC‐B dysregulates cytoskeleton assembly, trapping β‐catenin at osteoblast adhesion sites
Source: FEBS J. 2025 Jan 20;292(8):1920–33. doi: 10.1111/febs.17399 (PMC12001182; doi:10.1111/febs.17399)
Supplement: Supplementary file 1 — Fig. S1. Original, uncropped images of all western blots. Fig. S2. Negative controls for the immunofluorescence analyses. [file FEBS-292-1920-s001.pdf]

(A)

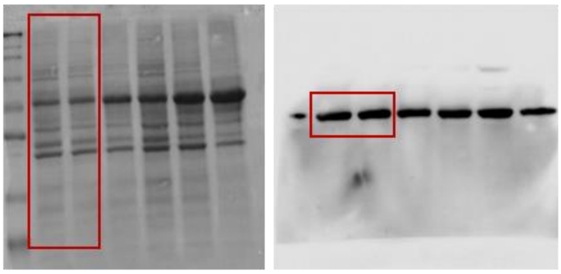

(B)

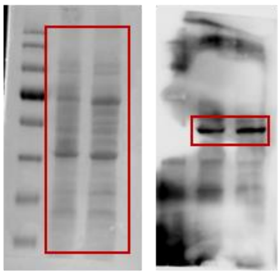

(C)

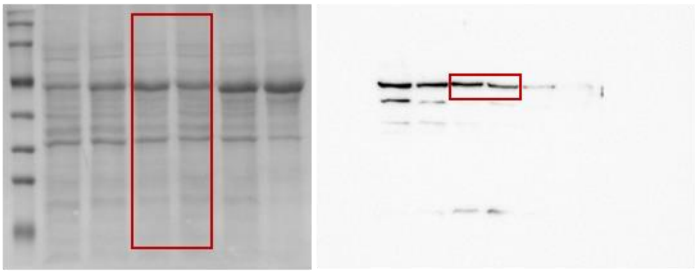

(D)

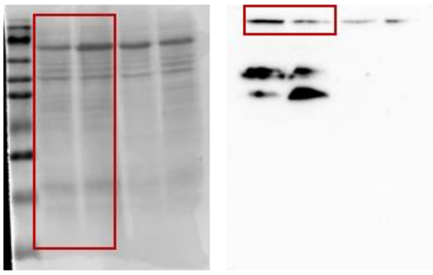

(E)

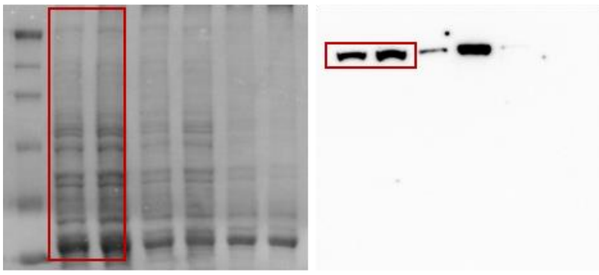

(F)

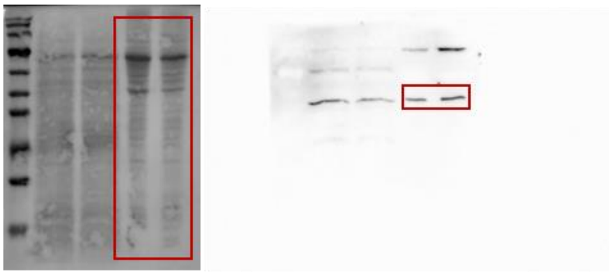

(G)

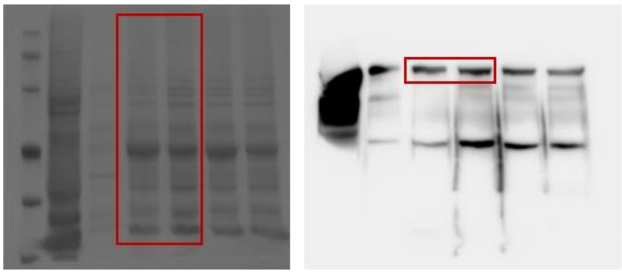

(H)

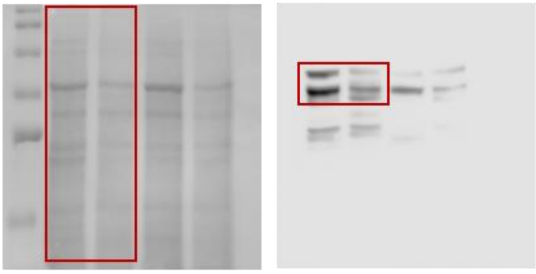

(I)

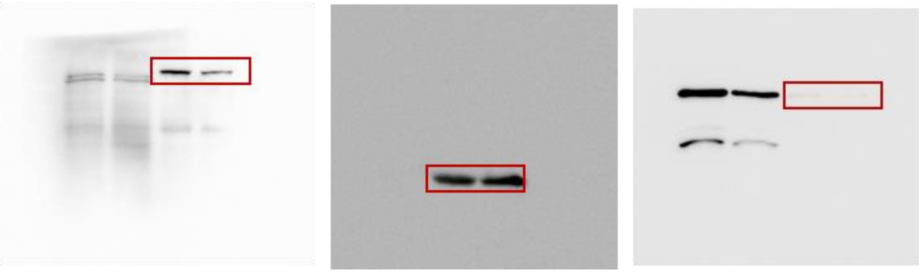

Figure S1: Original, uncropped images of all western blots.

Total protein staining was used to normalize the protein expression and is shown on the left of each panel. The red boxes show the cropped bands as presented in the main figures. Cropping has been done on the basis of the protein molecular weight. (**A**) β-actin as shown in Figure 1C, (**B**) fascin as shown in Figure 2A, (**C**) phospho-Fascin as shown in Figure 2C, (**D**) phospho-MARCKS as shown in Figure 2D, (**E**) phospho-FAK as shown in Figure 3C, (**F**) cleaved phospho-FAK as shown in Figure 3D, (**G**) total β-catenin as shown in Figure 4C, (**H**) Lamin A/C as shown in Figure 5F, (**I**) nuclear β-catenin/GAPDH/H3K9ac as shown in Figure 5B.

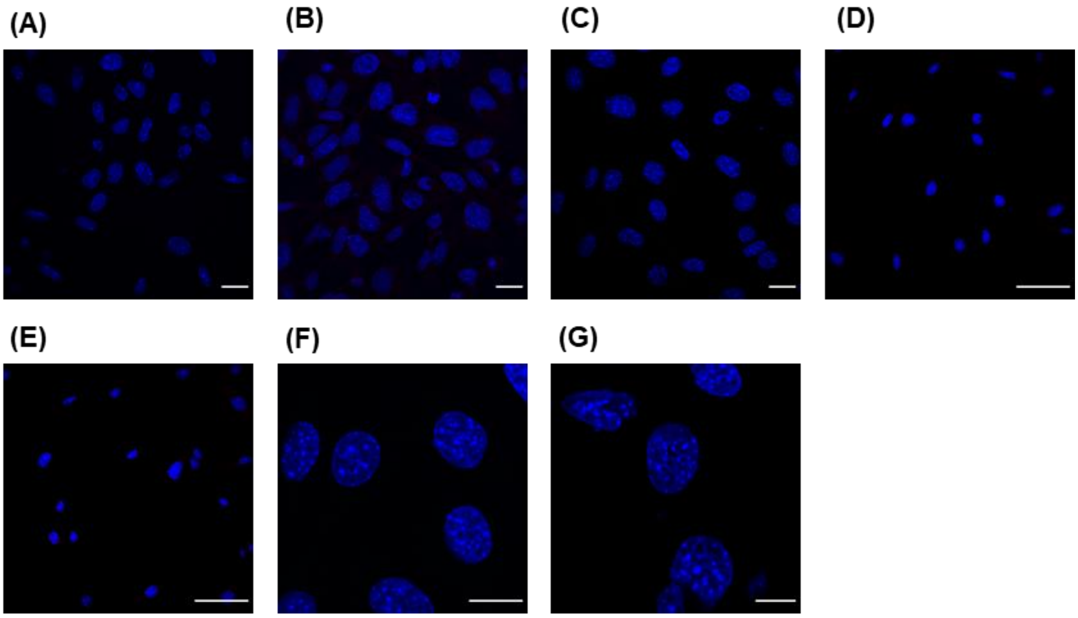

Figure S2: Negative controls for the immunofluorescence analyses.

Negative controls for the immunofluorescence analyses were obtained following the reported protocol but without incubation with the primary antibodies to confirm the specific antibodies signal. Cells were stained for (**A**) phalloidin (scale bar 20 μm), (**B**) fascin (scale bar 20 μm), (**C**) β-catenin (scale bar 20 μm), (**D**) E-cadherin (scale bar 40 μm), (**E**) N-cadherin (scale bar 40 μm), (**F**) nuclear β-catenin (scale bar 5 μm), (**G**) Lamin A/C (scale bar 5 μm).
